# Supplementary material for: In Vitro Evaluation of Antimicrobial Amyloidogenic Peptides for the Treatment of Early and Mature Bacterial Biofilms
Source: Int J Mol Sci. 2025 Sep 9;26(18):8767. doi: 10.3390/ijms26188767 (PMC12470154; doi:10.3390/ijms26188767)
Supplement: Supplementary file 1 [file ijms-26-08767-s001.zip › Supplementary S2.pdf]

Supplementary

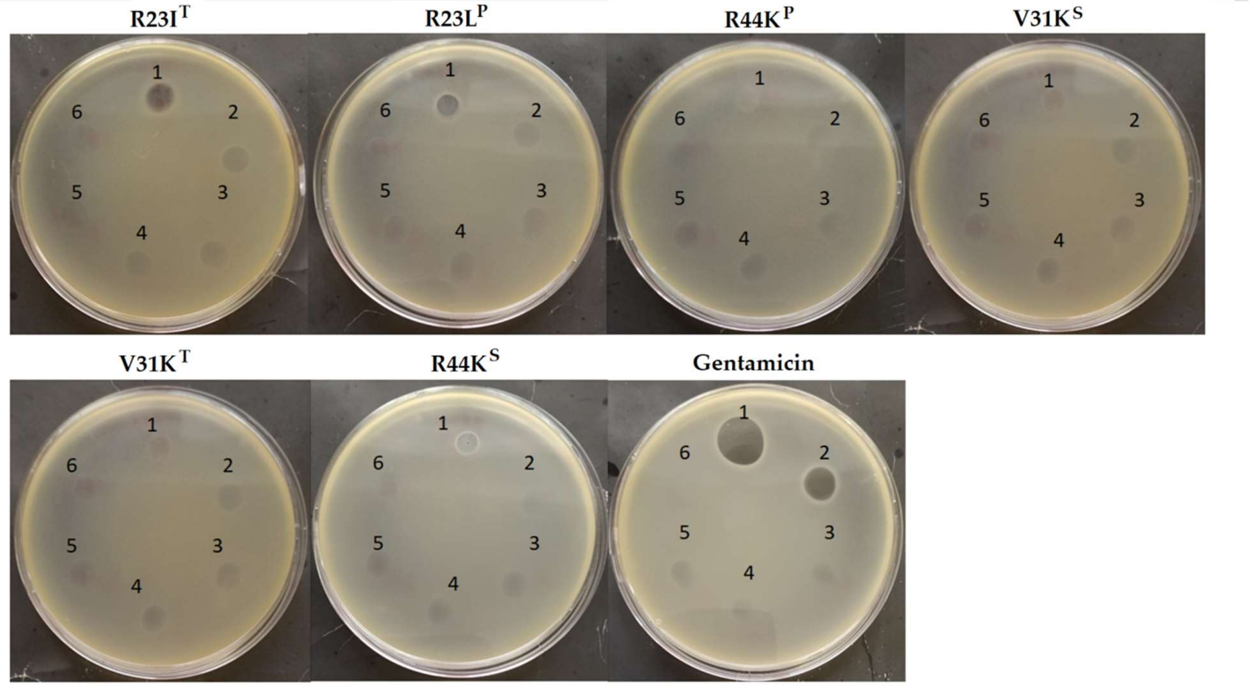

Figure S1. Antimicrobial effects of peptides against *S. aureus* MRSA SA180-F. 1, 2, 3, 4 – peptide samples at concentrations 1 mg/ml, 0.1 mg/ml, 0.01 mg/ml, and 0.001 mg/ml respectively; 5 – 20% DMSO; 6 – PBS.

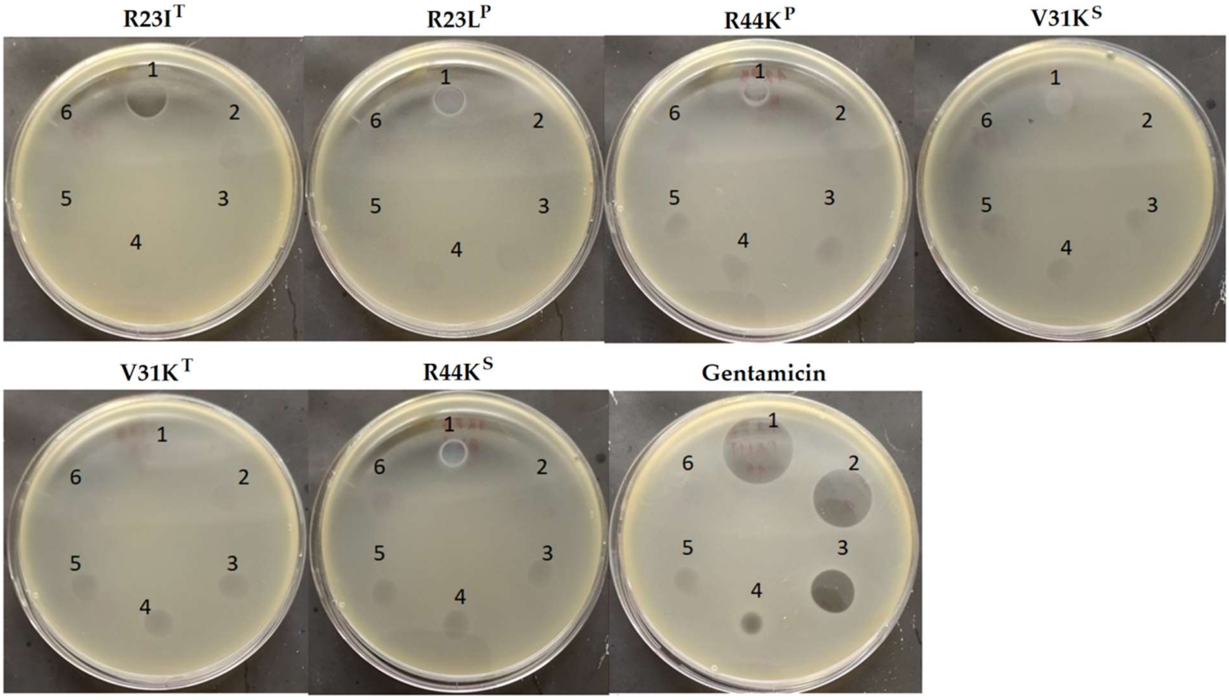

Figure S2. Antimicrobial effects of peptides against *S. aureus* 129B. 1, 2, 3, 4 – peptide samples at concentrations 1 mg/ml, 0.1 mg/ml, 0.01 mg/ml, and 0.001 mg/ml respectively; 5 – 20% DMSO; 6 – PBS.

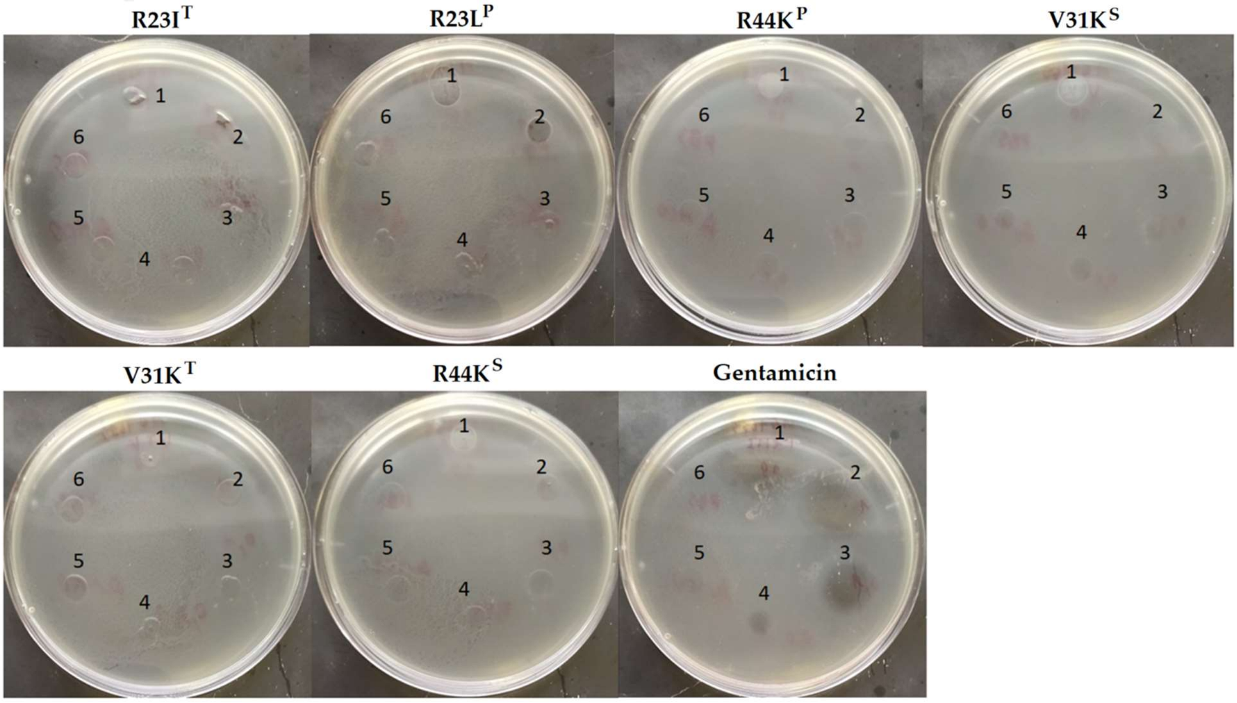

Figure S3. Antimicrobial effects of peptides against *E. coli* MG1655. 1, 2, 3, 4 – peptide samples at concentrations 1 mg/ml, 0.1 mg/ml, 0.01 mg/ml, and 0.001 mg/ml respectively; 5 – 20% DMSO; 6 – PBS.

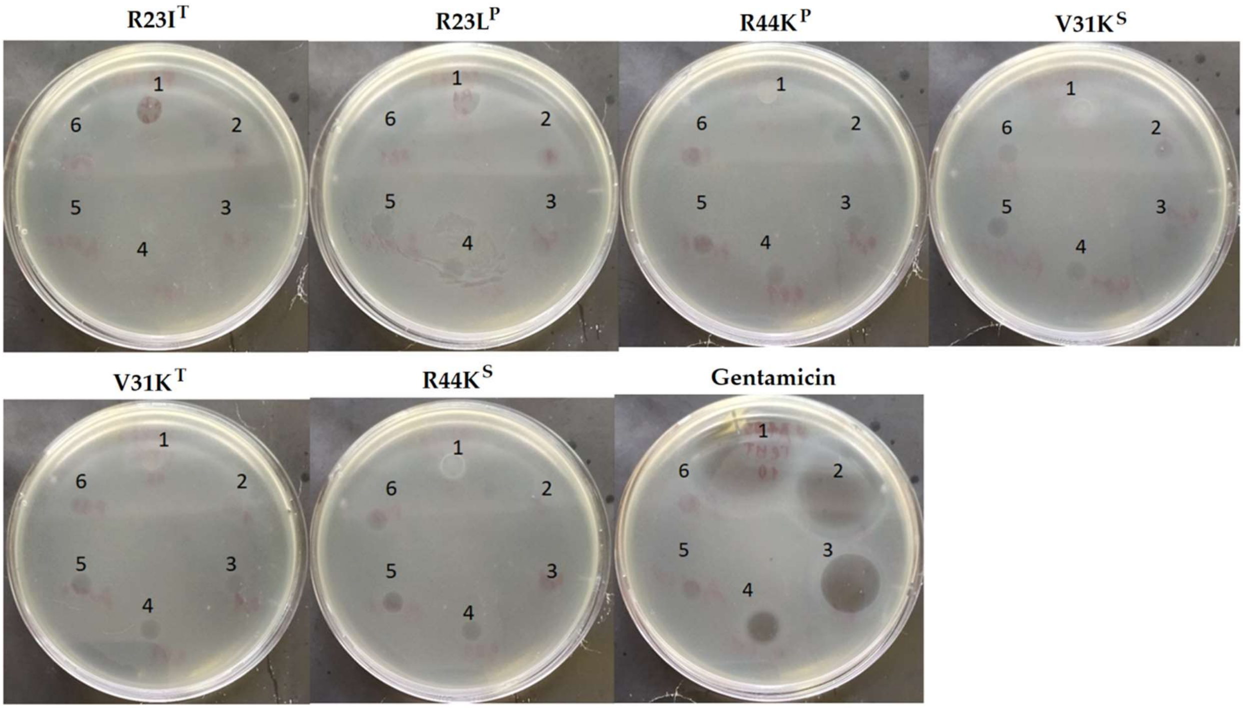

Figure S4. Antimicrobial effects of peptides against *P. aeruginosa* 2943. 1, 2, 3, 4 – peptide samples at concentrations 1 mg/ml, 0.1 mg/ml, 0.01 mg/ml, and 0.001 mg/ml respectively; 5 – 20% DMSO; 6 – PBS.
