# Supplementary material for: In Vitro Evaluation of Antimicrobial Amyloidogenic Peptides for the Treatment of Early and Mature Bacterial Biofilms
Source: Int J Mol Sci. 2025 Sep 9;26(18):8767. doi: 10.3390/ijms26188767 (PMC12470154; doi:10.3390/ijms26188767)
Supplement: Supplementary file 1 [file ijms-26-08767-s001.zip › Supplementary S3.pdf]

Table S1. Characteristics of the peptides from chromatography and mass spectrometry analysis

**R23I^{T}**	**RKKRRQRRRGGGGVTDFGVFVEI**
Peptide purity by HPLC	98.9 %
Calculated average mass	2675.1
Observed monoisotopic mass (MALDI-TOF MS)	2674.4
**R23L^{P}**	**RKKRRQRRRGGGGITDFGIFIGL**
Peptide purity by HPLC	99.6 %
Calculated average mass	2645.1
Observed monoisotopic mass (MALDI-TOF MS)	2644.5
**V31K^{T}**	**VTDFGVFVEIGGGGSRQIKIWFQNRRMKWKK**
Peptide purity by HPLC	94.52 %
Calculated average mass	3669.3
Observed monoisotopic mass (MALDI-TOF MS)	3667.7
**R44K^{S}**	**RKKRRQRRRGGGGVVVHINGGKFGGGGSRQIKIWFQNRRMKWKK**
Peptide purity by HPLC	98.3 %
Calculated average mass	5163.1
Observed monoisotopic mass (MALDI-TOF MS)	5163.2
**R44K^{P}**	**RKKRRQRRRGGGGITDFGIFIGLGGGGSRQIKIWFQNRRMKWKK**
Peptide purity by HPLC	94.54 %
Calculated average mass	5189.1
Observed monoisotopic mass (MALDI-TOF MS)	5189.4
**V31K^{S}**	**VVVHINGGKFGGGGSRQIKIWFQNRRMKWKK**
Peptide purity by HPLC	98.03 %
Calculated average mass	3613.3
Observed monoisotopic mass (MALDI-TOF MS)	3611.7
**I31K^{P}**	**ITDFGIFIGLGGGGSRQIKIWFQNRRMKWKK**
Peptide purity by HPLC	>95 %

Calculated average mass	3639.3
Observed monoisotopic mass (MALDI-TOF MS)	3637.8

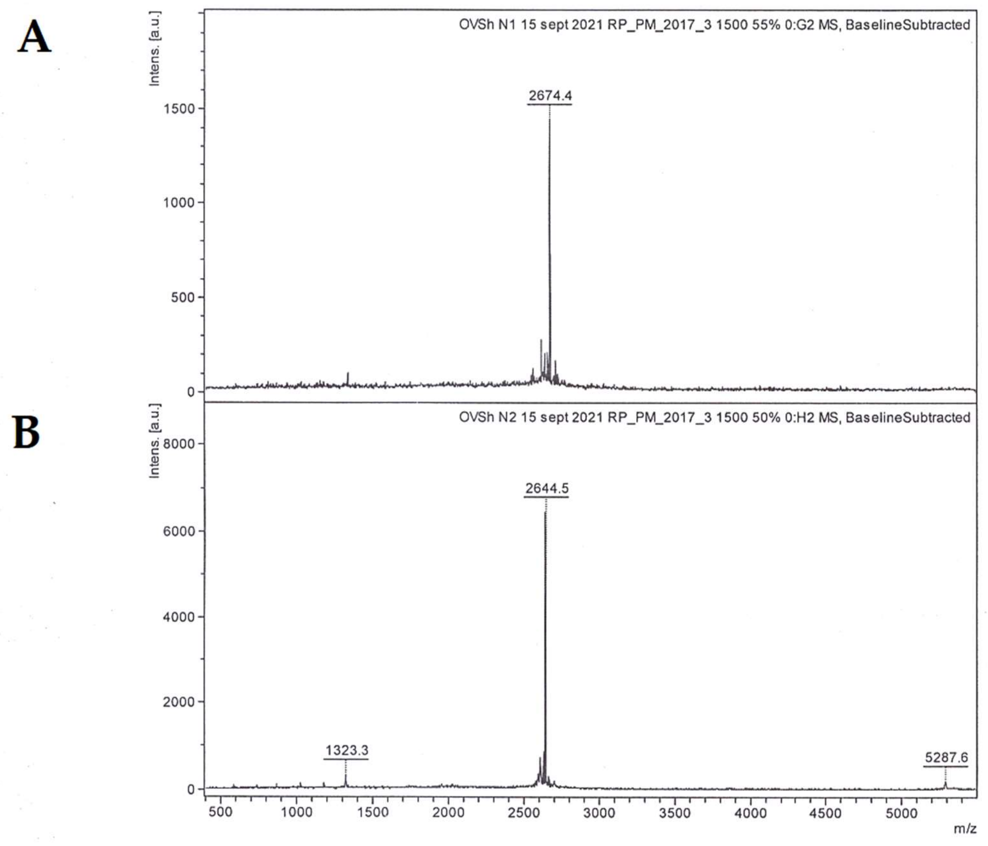

Figure S5. Mass spectrometry analysis R23I^{T} (**A**) and R23L^{P} (**B**).

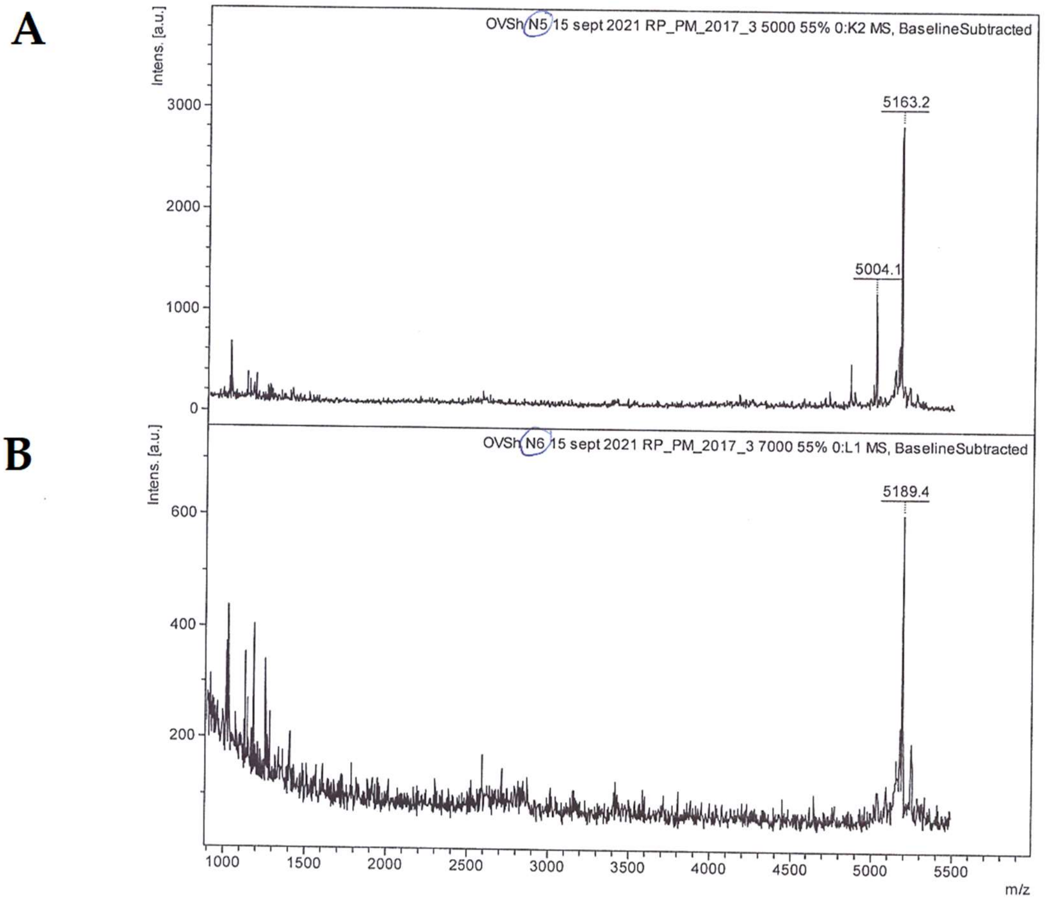

Figure S6. Mass spectrometry analysis R44K^S (**A**) and R44K^P (**B**).

A

B

C

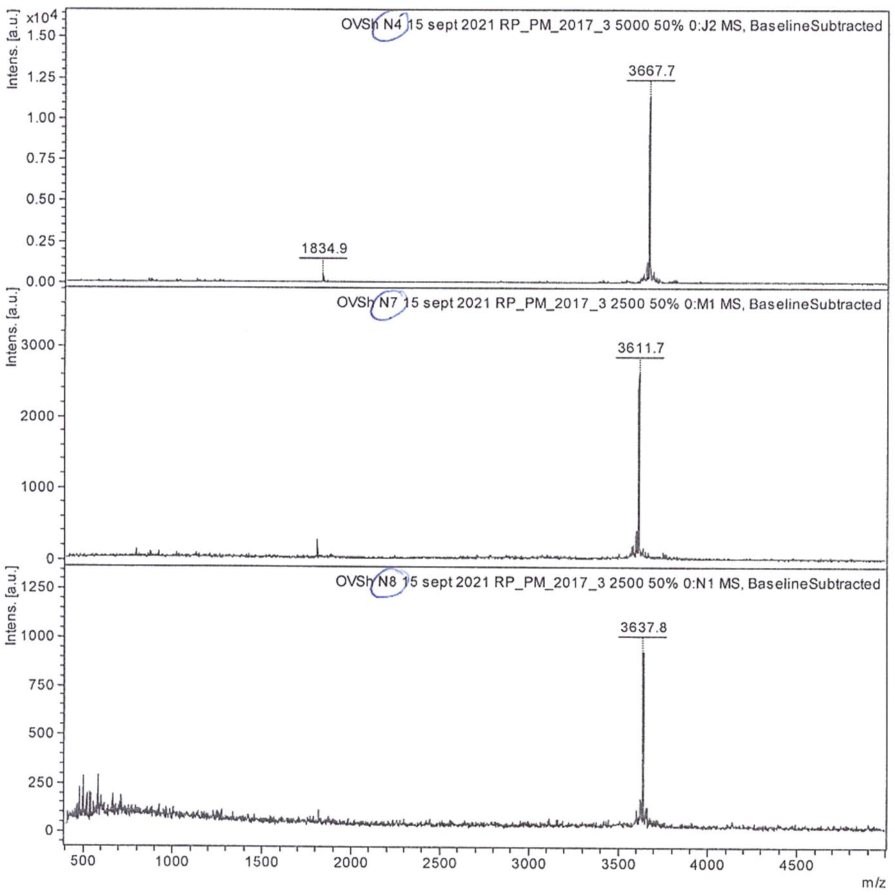

Figure S7. Mass spectrometry analysis V31K^{T} (**A**), V31K^{S} (**B**), and I31K^{P} (**C**).
